# Trends of Inequalities in Early Initiation of Breastfeeding in Ethiopia: Evidence from Ethiopian Demographic and Health Surveys, 2000-2016

**DOI:** 10.1155/2022/5533668

**Published:** 2022-02-27

**Authors:** Tsegaw Amare, Endalkachew Dellie, Getasew Amare

**Affiliations:** Department of Health Systems and Policy, Institute of Public Health, College of Medicine and Health Sciences, University of Gondar, Gondar, Ethiopia

## Abstract

**Background:**

Early initiation of breastfeeding (EIBF) is a costless practice with numerous neonates' survival benefits. Thus, any disparity results in an unacceptably high neonatal death rate but socioeconomic disparities on EIBF have not been well explored in Ethiopia. Therefore, this study is aimed at assessing the socioeconomic inequalities of EIBF in Ethiopia from 2000 to 2016.

**Methods:**

The Ethiopian demographic and health survey data and the World Health Organization's Health Equity Assessment Toolkit were used to investigate the inequalities in EIBF across the wealth quintile, education, residence, and subnational region. Difference, ratio, slope index inequality (SII), relative index inequality (RII), and population attributable risk (PAR) were used as equity summary measures.

**Results:**

In Ethiopia, EIBF practice was 47.4% in 2000, 66.2% in 2005, 51.5% in 2011, and 73.3% in 2016. Wealth-related inequality was observed in the 2000, 2005, and 2011 survey years with SII of -7.1%, -8.8%, and 8.7%, respectively, whereas educational-related inequality was observed in 2005 and 2011 with SII of -11.7% and 6.5%, respectively. However, significant change in wealth-, education-, and residence-related inequalities was detected in 2011. Regional inequality on EIBF was observed in all survey years with a difference of 35.7%, 38.0%, 29.1%, and 48.5% in the 2000, 2005, 2011, and 2016 survey years, respectively. But a significant change in regional inequality was noted in 2016 with a PAR of 17.2%.

**Conclusions:**

In Ethiopia, the wealth-, residence-, and educational-related inequalities of EIBF increased significantly between the years 2000 and 2011. However, regional inequality persistently increased from 2000 to 2016. Overall, one-sixth of the national level EIBF was decreased due to regional disparity in 2016. The northern regions of Ethiopia (Tigray, Afar, and Amhara) poorly performed compared to the peer regions. Therefore, interventions targeting them would significantly improve the national level of EIBF.

## 1. Introduction

Early initiation of breastfeeding (EIBF) is breastfeeding to newborns initiated within the first one hour of birth to benefit both the neonate and the mother [1]. The World Health Organization (WHO) recommends the EIBF to enrich the newborn with protective nutrients which are abundant in the first milk of breastfeeding and to facilitate the emotional bonding of the mother and the newborn which is in turn used for the physiological resilience of the mother in the postpartum period [2].

Globally, an estimated 22% of early neonatal deaths were attributed to the late initiation of breastfeeding in 2018 [3, 4]. The failure of EIBF in the first hour of birth is estimated to double the risk of neonatal death [5, 6]. Even though EIBF is a cost-effective and easy to implement action with significant implications for saving newborn and maternal lives, only 42% of mothers initiate breastfeeding within the first hour of birth worldwide [7]. The magnitude often varied globally, with high-income countries having a higher practice of EIBF than low- and middle-income countries [8].

Although EIBF in Ethiopia increased from 48.8% in 2000 to 75.7% in 2016 [9], it was far from the national target of 92% by 2020 [10]. The recent prevalence of EIBF was 83.7% in southern Ethiopia [11], 73.1% in the Dembecha district of Northwest Ethiopia [12], and 42.8% in Arbaminch of southern Ethiopia [13]. In addition, the 2010 report of the Federal Ministry of Health revealed the lowest EIBF practised in Amhara (38%) and Somali regions (40%) and the highest in the South Nation Nationality of People Region (SNNPR) (67%) and Dire Dawa regions (66%) [10].

Besides, evidence showed that the EIBF varied on the sex of the neonate [14–16], residence [17], educational status [17–19], region [16, 17, 19–21], and wealth status of the mother [15, 18, 20, 21]. Consistently, studies indicated mothers' age, mother's residence, educational status, wealth status, and the subnational region of the mother as the socioeconomic determinants of EIBF [11–14, 19, 22].

Though addressing the socioeconomic disparities in EIBF would have an impact on achieving the neonatal and maternal mortality sustainable goals [23], there is a scarcity of evidence in Ethiopia showing the trend on socioeconomic inequality in EIBF. In addition, as one of the preventive measures, identifying the gap in EIBF could influence the maternal mortality reduction that Ethiopia predicted not to achieve by 2030 [24]. On the other hand, assessing the disparity of EIBF across different dispersion measures would give complete evidence to design more specific and effective interventions.

Moreover, no study was conducted in Ethiopia using the WHO recommendation to assess the trend on socioeconomic inequality to have comprehensive evidence. The WHO recommends inequality to be measured using the absolute and relative measures by applying complex and simple dispersion measures for the selected health indicator to compare the disparities across the inequality dimensions. Hence, employing the WHO-recommended inequality measure would give impactful evidence by comparing fundamental inequality stratifies across the dimensions. Therefore, this study is aimed at assessing the trend on socioeconomic inequalities of EIBF in Ethiopia using the Ethiopian demographic and health surveys from 2000 to 2016.

## 2. Methods

### 2.1. Study Setting

Ethiopia is the second highly populated country in Africa, containing 116,831,357 inhabitants with a per capita income of US$850 in 2019 [25, 26]. For administrative purposes, Ethiopia has 11 regions, namely, Tigray, Amhara, Oromia, Southern Nation Nationalities and Peoples Region (SNNPR), Afar, Somalia, Gambela, Benishangul, Dire Dawa, Addis Ababa, and Harari. The country has a three-tiered healthcare system with its health policy prioritizing disease prevention with a special focus on maternal and child health [27]. The primary level includes the primary hospitals, the health centres, and the health posts in which essential and nonspecialized health services are provided. The secondary level contains the general hospitals that provide curative services, and the tertiary level consists of the comprehensive specialized hospitals that offer superspecialist care [27]. Besides, for the past two decades, the country implemented the health extension program to reach the highly remote areas and the rural residents of Ethiopia under the primary level of health care [28]. Though most maternal and child health services are exempted health services in Ethiopia [29], there are observed socioeconomic and area-based inequalities towards the uptake of maternal and child health services in favour of the advantageous subgroups [30, 31].

### 2.2. Study Design, Data Source, and Sampling Procedure

The secondary data used in this study were from four nationally representative cross-sectional Ethiopian Demographic and Health Surveys (EDHS) conducted in 2000, 2005, 2011, and 2016. These surveys provide data on key demographic and health indicators including maternal and child health.

The EDHS was collected using a two-stage stratified sampling technique. In the first stage, independent selection was employed in each sampling enumeration area after classifying the country into two enumeration areas with a proportional probability depending on the population size of the enumeration area. In the second stage of selection, a systematic selection of the newly created household listing from a fixed number of households per cluster was selected with an equal probability after a household listing operation was carried out in all selected enumeration areas. A total of 3680, 3528, 4037, and 3861 women aged 15 to 49 years who gave birth two years preceding 2000, 2005, 2011, and 2016 survey years, respectively, were used in this study [32–35].

### 2.3. Study Variables

Early initiation of breastfeeding was the outcome variable for which inequality was measured. According to the WHO definitions for assessing infant and young child feeding [36], EIBF was calculated as the ratio of women with live birth and puts their newborn to the breast within the first one hour of delivery to the total number of women with a live birth in the two years before the survey.

The inequality is disaggregated by educational status, place of residence, economic status, and subnational regions. Educational status was classified as no education, primary education, and secondary education and above. The economic status was categorized into five quintiles, from the poorest (quintile 1) to the richest (quintile 5) sequentially. The place of residence was classified as rural and urban, and the subnational regions included the nine regions and two city administrations. The place of residence and subnational region did not show up in the sequential presentation of the study participants. The trend on the socioeconomic inequality of EIBF was presented using tables and figures. The disaggregation included the computed point estimates with a corresponding 95% uncertainty interval (UI).

### 2.4. Data Analysis

The data were obtained as part of WHO's Health Equity Assessment Toolkit (HEAT) software [37]. The 2021 updated online version (version 4.0) of HEAT software was used for this study. More than 30 critical health indicators on reproductive, maternal, and child health were included in the updated version. Besides, six inequality dimensions (age, sex, economic status measured as wealth decile or wealth quintile, education, place of residence, and subnational region) were included to perform inequality assessment for more than 450 international household surveys conducted in 115 countries between 1991 and 2018. The HEAT software's essential purpose was to run country's health equity assessment and compare its trend over time and with other countries' inequality. The software allows to perform the summary measure of health inequality and segregate the data across the different dispersion measures. The HEAT software is a comprehensible, interactive, and easy-access software to compare health inequality [37].

The measure of inequality can be performed through relative and absolute inequality measures, which can be simple or complex [38, 39]. The criteria for selecting the type of measurement of inequality depend on the type of variable (ordering or nonordering) that the disparity is segregated. In this study, Difference (D), Ratio ®, Relative Index of Inequality (RII), Slope Index of Inequality (SII), and Population Attributable Risk (PAR) were used as a summary measure of dispersion for the EIBF trend in Ethiopia. These summary measures were selected due to their more comprehensive application to the inequality assessment [40–42].

“Difference” is the simple and absolute measure of inequality calculated as the mean percentage of EIBF in the one group subtracted from the mean percentage of EIBF in the other subgroup, whereas “Ratio” is the simple and relative measure of inequality calculated as the percentage of EIBF percentage in one subgroup to the mean percentage of EIBF in the other subgroup. The two main limitations of simple measures of inequality were the ignorance of the middle subgroups and not considering population size [39, 43].

On the other hand, “slope index inequality” is the complex and absolute measure of inequality that applies to natural ordering subgroups like education and wealth. It performs inequality measures by ranking from the disadvantaged subgroup to the advantageous subgroup and subtracting from the advantageous subgroup to the disadvantageous subgroup; thus, a positive value shows that the EIBF is more prevalent in the advantageous subgroup. The negative value shows the EIBF is more prevalent in disadvantageous subgroups. Besides, “relative index inequality” is a complex and relative measure of inequality determined by dividing the predicted EIBF from the highest rank to the lowest rank of the entire distribution for nonordering stratifies like urban, subnational region, and sex. The complex measure of inequality addresses the limitation of the simple measure of inequality by producing a single value expressing the disparity across the subgroups considering population's size [44].

Population attributable risk is the absolute measure of inequality that shows how much the disparity is eliminated by improving the EIBF in the population relative to the best-performing subgroup, keeping the improvement rate constant as the reference subgroup. It is calculated as the difference between the estimate for the reference subgroup and the national level [44].

The trend of EIBF was assessed across the four equity stratifies for each of the four survey years from 2000 to 2016 EDHS. The point estimate of the proportion of EIBF in each survey year was computed with the 95% uncertainty interval (UI). To declare a statistically significant disparity in Difference, SII, and PAR, the 95% UI should not include zero, and in Ratio and RII, the 95% UI should not include one. Whereas to declare a significant change in inequality over time, the UIs of the summary measure must not be overlapped [42]. Moreover, this paper was prepared according to the guideline for Strengthening the Reporting of Observational Studies in Epidemiology (STROBE) as a meant for logical and scientific representations of the study findings [45].

### 2.5. Ethical Considerations

This study does not need ethical clearance as the data were available publicly and uploaded as part of the WHO HEAT software. The institution that conducted the survey completed all the necessary ethical procedures. Besides, the Institutional Review Board of Ethiopia and the Inner-City Fund international approved the EDHS.

## 3. Results

### 3.1. The Proportion of EIBF Across Equity Dimensions and Survey Years

**Figure 1:**
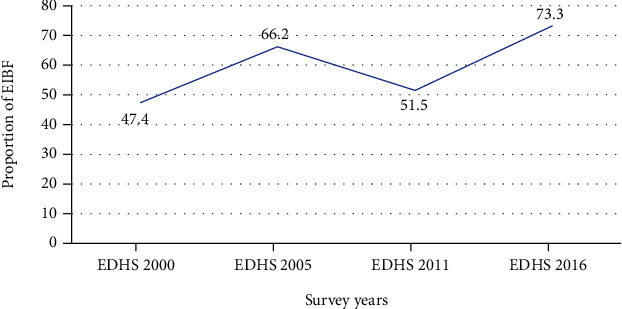
The trend of EIBF across the four rounds of the EDHS (2000 to 2016).

This study indicated a fluctuation in EIBF practice in Ethiopia for the last seventeen years, with 47.4%, 66.2%, 51.5%, and 73.3% in 2000, 2005, 2011, and 2016, respectively (Figure 1). The EIBF changed from 44.3% to 74.4% in the richest subgroups and 52.3% to 73.9% in the poorest subgroups. Unexpectedly, the EIBF decreased in both wealth quintiles in the 2011 survey year, from 69.9% to 48.5% in the poorest quintile, and 61.7% to 57.8% in the richest quintile.

In addition, EIBF practice enhanced persistently from 40.4% to 69.8% among the higher educated subgroups and 47.8% to 73.4% among noneducated subgroups. On the other hand, EIBF practice improved from 47.8% to 75.8% among rural residents and 44% to 78.3% among urban residents. Consistent with the wealth quintile, the EIBF practice decreased in both rural and urban residents in 2011 from 66.6% to 50.6% and from 61% to 57.1%, respectively. Furthermore, the EIBF practice persistently increased only in Addis Ababa and fluctuated in the rest of the subnational regions but was higher in 2016 than in the 2000 survey year in all regions (Table 1).

### 3.2. Early Initiation of Breastfeeding Inequalities Based on Different Summary Measures

**Figure 2:**
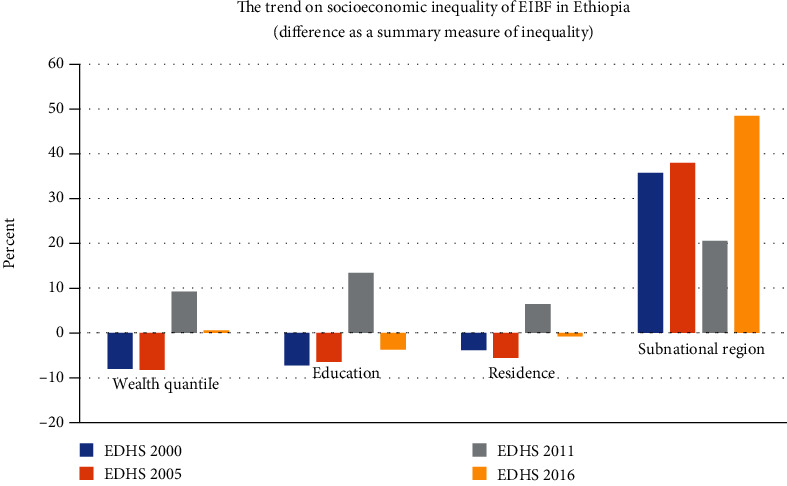
The trend on socioeconomic inequality of EIBF in Ethiopia (difference as a summary measure of inequality).

In Ethiopia, wealth-related inequality was observed in the 2000, 2005, and 2011 survey years with SII of -7.1%, -8.8%, and 8.7%, respectively. But the significant change in wealth-related inequality on EIBF was observed in 2011 with a PAR of 6.3%. Besides, educational-related inequality was observed in 2005 and 2011 with SII of -11.7% and 6.5%, respectively. Likewise, wealth inequality is a significant change in EIBF in education-related inequality observed in 2011 with a PAR of 12.6%. In addition, residence-related inequality was observed only in 2011 with a PAR of 5.6%. On the other hand, regional inequality on EIBF was observed in all survey years with a difference of 35.7%, 38.0%, 29.1%, and 48.5% in the 2000, 2005, 2011, and 2016 survey years, respectively. But a significant change in regional inequality was observed in 2016 with a PAR of 17.2% (Table 2) (Figure 2).

## 4. Discussion

This study is aimed at assessing the trend on socioeconomic inequalities of EIBF in Ethiopia using the WHO health equity assessment toolkit. Our finding showed that in Ethiopia, the EIBF practice fluctuated since 2000 but increased by 1.5 between 2000 and 2016. The increment rate in EIBF is in line with the rate of increment in Indonesia [20]. However, the finding is lower than the achievement in Ghana [46], where the proportion of EIBF increased by a factor of 3.0 from 1998 to 2014, and Bangladesh [14], where the rate of EIBF increased by a factor of 2.0 between the years 2004 and 2014. The difference might be due to the shorter duration of the trend observed in Bangladesh (10 years) than in Ethiopia (17 years) and might be due to the implementation of community mutual health organizations in Ghana that improved the overall health outcomes in the specified period [47].

In 2016, the magnitude of EIBF practice was 73.3% in Ethiopia and which is good based on the WHO classification of percentages of breastfeeding within 1 h after delivery as (0%–29%) poor, (30%–49%) fair, (50%–89%) good, and (90%–100%) [1]. The finding is also in line with sub-Saharan Africa countries' range, 17% to 82% [48]. However, it is higher than the practice in Economic Community of West African States [15], Ghana [46], Sudan [49], Guinea [50], Indonesia [20], Bangladesh [14], and India [17], where 43%, 55.1%, 69%, 60%, 57.29%, 51.24%, and 41.5% practice of EIBF, respectively. The discrepancy might be due to the health extension program application in Ethiopia's health care system. Health extension workers create awareness to pregnant mothers and follow them to the uptake of maternal health services, which includes the breastfeeding practice of the newborn. The result also remembers Ethiopia's achievement of the millennium development goal of child mortality reduction three years before the deadline [51]. On the other hand, the practice of EIBF in Ethiopia is lower than the 98.4% practice in Angola [52] and the inconsistency might be due to the absence of the WHO's highly recommended baby-friendly hospital initiative in Ethiopia [53–55].

In addition, the study showed that wealth-related inequality was observed in 2000, 2005, and 2011 survey years but the significant reverse change from favour to poorest subgroup to the favour of richest subgroup in wealth-related inequality on EIBF was observed in 2011 with the richest subgroups practice with a weighted difference of 8.7% higher than the poorest subgroups. In the same year, wealth-related inequality was attributable to a 6.3% decrement in the national EIBF magnitude, whereas educational-related inequality was observed in 2005 and 2011 with noneducated subgroups practising a weighted difference of 11.7% higher than the higher educated subgroups. But it changed significantly and reversely to a 6.5% increment in the educated subgroups than in the noneducated subgroups in 2011. If educational inequality on EIBF was made insignificant, the national level EIBF magnitude would have increased by 12.6% in 2011. Moreover, residence-related inequality was observed only in 2011 and which contributed to the 5.6% decline in national level EIBF practice in 2011.

However, wealth-, educational-, and residence-related inequalities disappeared in the 2016 survey year. The drastic change might be due to the implementation of the three-tier system in Ethiopia between 2010 and 2015 that gave a special emphasis and high priority to maternal and child health at primary health care units, where less-educated, poor, and rural residents were found. In addition, it might be due to the Alive and Thrive project implemented in Ethiopia between 2009 to 2014. The project used mass communication for the EIBF and optimal breastfeeding messages to families with children in the preceding two years and was 80% successful [56].

Furthermore, regional inequality on the EIBF was observed in all survey years. The difference between the highest and the lowest-performing region was 35.7% (64.1% in Harari and 28.4% in Tigray) in 2000, 38.0% (90.3% in Dire Dawa and 52.2% in Tigray) in 2005, 29.1% (66.5% in SNNPR and 37.5% in Amhara) in 2011, and 48.5% (90.5% in Dire Dawa and 42.0% in Afar) in 2016. The finding is consistent with the 2010 report of the Federal Ministry of Health which revealed that the lowest EIBF practice was observed in Amhara (38%) and the highest in the SNNPR (67%) [10]. The finding is consistent the evidence that shows regional disparity in the uptake of maternal and reproductive health services in Ethiopia [57]. The regional variation in EIBF was also observed elsewhere [20, 46, 50]. But a significant deterioration of regional inequality was observed in 2016 which is attributable to the 17.2% decrease in the national EIBF practice. The disparity might be due to program's implementation difference that the northern regions of Ethiopia are the most unstable areas during the political transition made over the period that may disrupt the implementation of maternal and child health programs. In addition, the drought and famine faced in the northern part of the country during the survey periods might also contribute for the poor breastfeeding practice in the northern Ethiopia.

The study has limitations that need to be considered while interpreting the results of the study. It should be noted the fact that the analysis in the current study is unable to reflect the current level of EIBF utilization and disparities in utilization rather owing to the time of data generation and should be interpreted only to the time when the data was generated. Besides, a retrospective data collection used in this study might be prone to recall bias. In addition, due to the nature of the toolkit, multivariate analysis was not conducted in this study, but inequality was discussed over different summary measures that helped to generate evidence.

## 5. Conclusion

In Ethiopia, the wealth-, residence-, and educational-related inequality of EIBF increased significantly between the years 2000 and 2011 but disappeared in the 2016 survey year. However, regional inequality has persistently increased for the past seventeen years. The significant change in wealth-, residence-, and educational-related inequality on the EIBF was detected in the 2011 survey years. On the other hand, regional inequality on the EIBF worsened in the 2016 survey year. The northern part of Ethiopia (Tigray, Afar, and Amhara) performed less than other regions in all survey years. Overall, one-sixth of the national level magnitude of the EIBF decreased due to regional disparity. Therefore, interventions targeting those regions would significantly improve the national level performance of EIBF for the subsequent reduction of neonatal mortality. In addition, regional variation should be researched further with advanced models like spatial analysis, and multivariate decomposition analysis should be done to identify the important determinants.

## Figures and Tables

**Table 1:** The trend of early initiation of breastfeeding by different inequality dimensions in Ethiopia (EDHS 2000–2016).

Dimensions	Years											
	2000			2005			2011			2016		
	Estimate	LB	UB	Estimate	LB	UB	Estimate	LB	UB	Estimate	LB	UB
Wealth quintile												
Quintile 1 (poorest)	52.3	47.6	57.1	69.9	65.1	74.3	48.5	43.2	54.0	73.9	69.1	78.1
Quintile 2	48.7	44.3	53.3	66.8	62.0	71.3	50.9	46.0	55.9	75.6	70.6	79.9
Quintile 3	42.0	37.2	47.0	66.5	61.9	70.8	50.7	45.8	55.6	73.4	69.0	77.4
Quintile 4	49.4	44.2	54.6	64.6	59.7	69.2	51.4	45.1	57.6	69.0	63.3	74.1
Quintile 5 (richest)	44.3	39.6	49.1	61.7	55.3	67.7	57.8	52.1	63.2	74.4	68.9	79.3
Educational status												
No education	47.8	44.9	50.7	67.5	64.5	70.4	50.7	46.9	54.4	73.4	70.6	76.1
Primary education	47.8	41.8	53.8	61.6	56.6	66.3	51.5	46.8	56.2	74.1	70.0	77.9
Secondary education and above	40.4	32.0	49.5	61.1	52.4	69.1	64.1	54.9	72.3	69.8	62.9	75.9
Residence												
Rural	47.8	44.9	50.6	66.6	63.8	69.3	50.6	47.1	54.1	73.4	70.9	75.8
Urban	44.0	38.4	49.7	61.0	52.0	69.3	57.1	50.3	63.7	72.6	66.0	78.3
Subnational region												
Tigray	28.4	21.7	36.2	52.2	45.9	58.5	44.7	38.1	51.5	63.0	56.6	69.0
Afar	30.5	22.7	39.7	82.4	74.0	88.5	59.6	52.7	66.1	42.0	34.7	49.7
Amhara	28.4	22.9	34.7	59.8	53.9	65.5	37.5	31.2	44.2	66.0	59.7	71.8
Oromia	58.4	54.6	62.1	67.8	62.4	72.7	52.6	46.6	58.5	76.7	72.9	80.1
Somalia	50.3	33.7	66.7	85.4	78.4	90.5	39.6	31.2	48.5	78.2	71.7	83.5
Benishangul	46.7	36.3	57.4	68.7	58.3	77.5	42.2	33.0	52.1	71.7	66.0	76.8
SNNPR	54.1	48.1	60.0	69.8	65.8	73.6	66.5	61.0	71.6	77.1	71.7	81.7
Gambela	47.7	39.3	56.3	69.1	59.5	77.2	59.3	46.6	70.8	67.1	60.7	73.0
Harari	64.1	58.6	69.2	73.0	60.3	82.8	64.6	57.5	71.2	89.4	84.5	92.9
Addis Ababa	48.3	42.7	53.9	59.1	49.8	67.7	62.0	54.3	69.0	67.5	60.5	73.8
Dire Dawa	46.1	37.3	55.1	90.3	86.5	93.0	66.0	60.1	71.4	90.5	86.5	93.5
Total	*47.4*			*66.2*			*51.5*			*73.3*		

LB: Lower Bound; UB: Upper Bound; SNNPR: Southern Nations Nationalities and People's Region.

**Table 2:** Trends of early initiation of breastfeeding in Ethiopia based on different summary measures of inequality (EDHS 2000–2016).

Dimensions	Measure of inequality	Years											
		2000			2005			2011			2016		
		Estimate	LB	UB	Estimate	LB	UB	Estimate	LB	UB	Estimate	LB	UB
Wealth quintile	D	-*8.0*	-*14.8*	-*1.3*^∗^	-*8.2*	*-16.0*	*-0.5* ^∗^	*9.2*	*1.5*	*17.0* ^∗∗^	0.6	-6.3	7.5
	R	0.8	0.7	1.0	0.9	0.8	1.0	1.2	1.0	1.4	1.0	0.9	1.1
	RII	0.9	0.8	1.0	*0.9*	*0.8*	*0.9* ^∗^	*1.2*	*1.1*	*1.3* ^∗^	1.0	0.9	1.0
	SII	-*7.1*	-*12.2*	-*2.0*^∗^	-*8.8*	-*13.8*	*-3.9* ^∗^	*8.7*	*3.5*	*13.9* ^∗∗^	-3.0	-7.7	1.6
	PAR	0.0	-2.9	2.9	0.0	-2.7	2.7	*6.3*	*3.6*	*8.9* ^∗∗^	1.1	-1.3	3.5

Education	D	-7.3	-16.6	2.0	-6.5	-15.4	2.4	*13.4*	*3.9*	*22.9* ^∗∗^	-3.7	-10.7	3.4
	R	0.8	0.7	1.1	0.9	0.8	1.0	*1.3*	*1.1*	*1.5* ^∗∗^	1.0	0.9	1.1
	RII	0.9	0.8	1.1	*0.8*	*0.8*	*0.9* ^∗^	1.1	1.0	1.3	1.0	0.9	1.1
	SII	-4.6	-11.9	2.8	*-11.7*	*-18.3*	*-5.2* ^∗^	*6.5*	*0.3*	*12.6* ^∗∗^	-1.5	-6.8	3.7
	PAR	0.0	-0.7	0.7	0.0	-0.8	0.8	*12.6*	*11.5*	*13.6* ^∗∗^	0.0	-1.1	1.1

Residence	D	-3.8	-10.2	2.5	-5.6	-14.7	3.5	6.5	-1.1	14.1	-0.8	-7.4	5.8
	R	0.9	0.8	1.1	0.9	0.8	1.1	1.1	1.0	1.3	1.0	0.9	1.1
	PAR	0.0	-0.5	0.5	0.0	-0.4	0.4	5.6	5.0	6.2^∗∗^	0.0	-0.5	0.5

Subnational regions	D	*35.7*	*26.8*	*44.6* ^∗^	*38.0*	*31.0*	*45.1* ^∗^	*29.1*	*20.6*	*37.5* ^∗^	*48.5*	*40.2*	*56.8* ^∗∗^
	R	*2.3*	*1.7*	*2.9* ^∗^	*1.7*	*1.5*	*2.0* ^∗^	*1.8*	*1.5*	*2.1* ^∗^	*2.2*	*1.8*	*2.6* ^∗^
	PAR	*16.7*	*11.4*	*22.0* ^∗^	*24.1*	*18.1*	*30.0* ^∗^	*15.0*	*12.3*	*17.7* ^∗^	*17.2*	*2.2*	*32.2* ^∗^

^∗^Significant inequality. ^∗^Significant change in inequality with 95% UI; D: Difference; R: Ratio; RII: Relative Index of Inequality; SII: Slope Index of Inequality; PAR: Population Attributable Risk; LB: lower bound; UB: upper bound.

## Data Availability

The datasets supporting this article's conclusions are available online as part of the WHO health monitoring database. The DHS data can be acquired online from the DHS database through formal request available at https://dhsprogram.com/.
